# A Pilot Study: The Effect of CPAP Intervention on Sleep Architecture and Cognition in Alzheimer’s Disease Patients with Obstructive Sleep Apnea

**DOI:** 10.3390/neurolint17090147

**Published:** 2025-09-11

**Authors:** Carmen L. Frias, Marta Almeria, Judith Castejon, Cristina Artero, Giovanni Caruana, Andrea Elias-Mas, Karol Uscamaita, Virginia Hawkins, Nicola J. Ray, Mariateresa Buongiorno, Natalia Cullell, Jerzy Krupinski

**Affiliations:** 1 Fundació per a Docència I Recerca, MútuaTerrassa, 08221 Terrassa, Spain; 2 Department of Neurology, Fundació Assistencial Mútua Terrassa, 08221 Terrassa, Spain; 3 Neurology Department, Adsalutem Institute Sleep Medicine, 08008 Barcelona, Spain; 4 Neurology Service, Sleep Disorders Unit, Hospital Universitari Sagrat Cor, Grupo Quirónsalud, 08029 Barcelona, Spain; 5 Department of Psychology, Brooks Building, Faculty of Science and Education, Manchester Metropolitan University, Manchester M1 5GD, UK; 6 Neurology Department, Vall d’Hebron University Hospital, 08035 Barcelona, Spain; 7 Neurovascular Diseases Research Group, Vall d’Hebron Research Institute, 08035 Barcelona, Spain

**Keywords:** Alzheimer’s disease, cognitive decline, CPAP, mild cognitive impairment, obstructive sleep apnea

## Abstract

Background: Obstructive sleep apnea (OSA) is highly prevalent in the early stages of Alzheimer’s disease (AD), and its hallmark, sleep fragmentation, may accelerate cognitive decline. Continuous positive airway pressure (CPAP) improves OSA-related hypoxia during slow-wave sleep, but its cognitive benefits in AD remain unclear. Methods: We performed a 12-month sub-analysis of a prospective, longitudinal pilot study that enrolled 21 adults (median age = 77 yr; 71% women) with Mild Cognitive Impairment (MCI) with AD confirmed biomarkers and polysomnography-diagnosed OSA. All participants underwent baseline overnight polysomnography (PSG) and neuropsychological testing (Clinical Dementia Rating (CDR), Mini-Mental State Examination (MMSE), Repeatable Battery for the Assessment of Neuropsychological Status (RBANS)) that were repeated after 12 months. Twelve participants were CPAP-compliant (moderate/severe OSA) and nine were non-users (mild OSA/intolerance). Cognitive change scores (Δ = 12 months -baseline) were compared with Generalized Linear Models (GLM) adjusted for baseline cognition and Apnea–Hypopnea Index (AHI); associations between baseline sleep parameters and cognitive trajectories were examined. And the association of sleep variables with the use of CPAP was also evaluated. Results: Compared with non-users, CPAP users showed significantly slower global decline (Δ MMSE: *p* = 0.016) and improvements in overall cognition (Δ RBANS Total: *p* = 0.028) and RBANS sub-domains (Δ RBANS FC: *p* = 0.010; Δ RBANS SF: *p* = 0.045). Longer baseline non-rapid eye movement (NREM) stage 3 and rapid eye movement (REM) sleep, greater total sleep time and sleep efficiency, and right-side sleeping were each linked to better cognitive outcomes, whereas extended NREM stage 2, wakefulness, and supine sleeping were associated with poorer trajectories. Conclusions: Twelve months of CPAP use was associated with attenuated cognitive decline and domain-specific gains in AD-related MCI with OSA. Sleep architecture and body position during sleep predicted cognitive outcomes, underscoring the therapeutic relevance of optimizing breathing and sleep quality. Larger, longer-term trials are warranted to confirm CPAP’s disease-modifying potential and to clarify the mechanistic role of sleep in AD progression.

## 1. Introduction

Alzheimer’s disease (AD) is the most prevalent neurodegenerative disorder, accounting for approximately 60–70% of dementia cases globally, and is characterized by progressive cognitive decline, memory impairment, and behavioral disturbances [1,2]. The disease manifests through the accumulation of amyloid-beta (Aβ) plaques and neurofibrillary tangles of hyperphosphorylated tau protein in the brain, leading to synaptic dysfunction, widespread neuronal death, and atrophy, particularly in regions crucial for memory and cognitive function [3,4,5]. Despite extensive research and significant advances in the understanding of the molecular and cellular mechanisms underlying AD, there remains no cure, and current treatments focus on symptom management rather than addressing the root causes of the disease [3,6,7].

The prodromal stage of AD is characterized by the presence of Mild Cognitive Impairment (MCI), which consists of cognitive decline that does not interfere significantly with daily life activities [8,9]. This is a critical period for early intervention, as it represents the initial stage before onset of dementia [7,8,9]. Individuals with MCI have annual conversion rates to AD ranging from 10 to 15% [4].

One of the most significant recent advances in neurobiology has been the discovery of the glymphatic system, a brain-wide network responsible for clearing metabolic waste products from the central nervous system (CNS), including Aβ [3,10,11]. This process is crucial for preventing the accumulation of Aβ and other toxic proteins that are implicated in the pathogenesis of AD [12,13]. Glymphatic transport is not uniform across the brain. In those regions with high metabolic activity and vulnerability to AD pathology, such as the hippocampus, medial temporal lobe, posterior cingulate/precuneus, and prefrontal cortex, exhibit both high demand for waste clearance and sensitivity to clearance failure. Impaired glymphatic exchange in these regions accelerates local accumulation of Aβ and tau, exacerbating neurodegeneration, and contributing to the progression of AD [14,15]. The glymphatic system is highly dependent on the sleep–wake cycle, with its activity probably being most pronounced during slow-wave sleep (SWS) phase of non-rapid eye movement (NREM) sleep characterized by low-frequency, high-amplitude delta waves [12,13]. Mechanistically, this enhancement during SWS is driven by a reduction in locus coeruleus noradrenaline (NE) output, which permits expansion of the interstitial and perivascular spaces, thereby increasing cerebrospinal fluid (CSF) penetrance into the parenchyma. NE levels during N3, NREM stage, also display slow oscillations, coupled with large-amplitude delta waves and vascular pulsations, which together facilitate CSF-interstitial fluid exchange [12,14]. Acetylcholine (ACh) tone is low during SWS, enabling the generation of synchronized slow waves; peripheral vagal effects on vascular tone, mediated by ACh, may indirectly support cerebral perfusion. However, the dominant role in glymphatic regulation is caused by NE. This state dependence explains why sleep disruption impairs glymphatic clearance. Studies have also shown that the efficacy of the glymphatic system declines with age, which, coupled with sleep disturbances common in older adults, may contribute to the increased risk of AD and accelerate its progression observed in the aging population [11,12].

Sleep plays a fundamental role in cognitive health, with disturbances in sleep being increasingly recognized as both a symptom and a contributing factor to neurodegenerative diseases like AD [2,10,16]. Sleep disturbances, including insomnia, fragmented sleep, and Obstructive Sleep Apnea (OSA), are prevalent among older adults and particularly in individuals with AD [16]. OSA is one of the sleep alterations with higher prevalence in AD, affecting between 43% and 91% of patients [16,17]. It is characterized by repeated episodes of partial or complete obstruction of the upper airway during sleep [16,18]. OSA leads to intermittent hypoxia, hypercapnia, frequent arousals, and significant fragmentation of sleep, particularly reducing the duration and quality of SWS [16,17,18]. OSA is also a potential cause of cognitive impairment, likely due to multiple potential mechanisms, including oxidative stress, increased inflammation, and the presence of cerebral small-vessel lesions [19]. This underscores the current significance of medical treatments.

Continuous positive airway pressure (CPAP) therapy is the gold standard treatment for OSA and has been proven to be effective in improving sleep quality by preventing airway collapse during sleep [17,18,20]. It increases the duration and quality of SWS, potentially enhancing the glymphatic system’s function and reducing the risk of Aβ accumulation in the brain [17,20]. Several studies have demonstrated that CPAP adherence in patients with OSA and MCI/AD can normalize sleep breathing and possibly lead to better cognition, which specific cognitive domains still need to be determined [17]. Other studies have also reported that CPAP could increase CSF flow rate in anesthetized rats, suggesting a possible therapeutic enhancement of glymphatic function [20].

Given the significant overlap between the prevalence of sleep disturbances in AD, as well as the potential for CPAP therapy to mitigate cognitive decline, this study aimed to explore the intricate relationship between OSA and MCI progression. We found that patients diagnosed with MCI and OSA with good CPAP adherence had improved sleep parameters and cognitive decline outcomes at 12-month follow-up, compared to those that did not use CPAP devices.

## 2. Methods

### 2.1. Study Design and Participants Inclusion

The current study consists of a sub-analysis of a prospective interventional and longitudinal pilot study. We included participants with MCI referred to the Cognition and Behaviour Unit at the Department of Neurology, Hospital Universitari MútuaTerrassa (HUMT) in Terrassa, Barcelona, Spain. Ethical approval from the Drug Research Ethics Committee of Fundació Assistencial Mútua Terrassa (approval code: P/21-132) was obtained on 17 November 2021. Participants also provided signed informed consent.

Participants with a diagnosis of MCI (confirmed by a Delayed Memory Index (DMI) below 85 on the Repeatable Battery for the Assessment of Neuropsychological Status (RBANS), a Mini-Mental State Examination (MMSE) score below 27, or a Clinical Dementia Rating (CDR) score of at least 0.5) and sufficient reading and writing skills to complete cognitive tests were included. AD pathology was confirmed by positive CSF AD biomarkers or positive 18F Flutemetamol PET/CT. AD biomarkers in CSF were measured by Catlab according to the established local cut-off values. Patients were considered positive for AD biomarkers when Aβ1-42/Aβ1-40 was <0.068 pg/mL plus two more of the following: t-tau (>404 pg/mL), p-tau 181 (>52.1 pg/mL), Aβ1-42 (<638 pg/mL), t-tau/Aβ1-42 (>0.784). For the current study, all the patients were required to have a sleep registry with overnight video-polysomnography (V-PSG).

Exclusion criteria included a prior diagnosis of other neurocognitive disorders, a history of affective disorders or psychosis, current participation in cognitive training programs, or the use of psychotropic medications affecting cognition. Those with a history of cerebrovascular accidents, transient ischemic attacks, or traumatic brain injury, as well as individuals with conditions likely to interfere with the study procedures, were also excluded.

### 2.2. Cognitive Assessment

Baseline cognitive assessments with CDR, MMSE, and RBANS tests were conducted, followed by a re-evaluation after 12 months.

CDR assesses the following areas: Memory, Orientation, Judgment and Problem Solving, Community Affairs, Home and Hobbies, and Personal Care. It generates a semi-quantitative and categorical score that classifies different levels of cognitive impairment, with higher scores indicating more severe impairment.

MMSE consists of five parts: Orientation, Registration, Attention, Calculation, Recall and Language. Each section contains questions that contribute to determining the cognitive impairment score.

RBANS is composed of five indexes, which are Immediate Memory Index (IMI), Visuospatial Index (VSPI), Language Index (LNGI), Attention Index (ATI), and Delayed Memory Index (DMI). To determine the value of each index, different subtests are also considered. IMI: List Learning (LL) and Story Memory (SM); VSPI: Figure Copy (FC) and Line Orientation (LO); LNGI: Picture Naming (PN) and Semantic Fluency (SF); ATI: Direct Digit Span (DDS), Indirect Digit Span (IDS) and Coding (C); DMI: List Recall (LR), List Recognition (LRe), Story Recall (SR) and Figure Recall (FR). To minimize learning effects, a parallel version of the same cognitive battery was used (version A at baseline and version B at follow-up). In both the MMSE and RBANS, lower scores indicate more severe cognitive impairment.

### 2.3. Video-Polysomnography (V-PSG) and Sleep Architecture

Participants underwent overnight V-PSG at Adsalutem sleep unit, where one sleep night was recorded using a comprehensive setup including synchronized audiovisual recording, electroencephalography (EEG; F3, F4, C3, C4, O1, and O2, referred to the combined ears), electrooculography (EOG), electrocardiography, surface electromyogram (EMG) of the right and left anterior tibialis in the lower limbs. Nasal cannula, nasal and oral thermistors, thoracic and abdominal strain gauges, and finger pulse oximeter were used to measure the respiratory variables. Sleep stages and respiratory events were scored according to the American Academy of Sleep Medicine’s Manual for Scoring of Sleep and Associated Events, Version 3. All participants from the current sub-study were diagnosed with OSA. The OSA’s severity was established from the Apnea–Hypopnea Index (AHI), extracted from the V-PSG, considering the following limits: Mild OSA (AHI: 5–15 events/hour), Moderate OSA (AHI: 15–30 events/hour) and Severe OSA (AHI: >30 events/hour) [21].

From V-PSG, we obtained the following sleep parameters: Duration of sleep stages, composed of non-rapid eye movement (NREM), sleep stages N1, N2 and N3; and duration of rapid eye movement (REM) sleep, total time in bed (total amount of time spent in bed including both sleep and wake periods), total sleep time (amount of time spent sleeping), sleep efficiency (ratio of the total sleep time to the time in bed, that could be affected by sleep disruptions) and wakefulness (amount of time spent awake while in bed), sleep position, including supine prone, right and left-side times. All these variables are measured in minutes, except sleep efficiency, which is indicated in percentage (%).

Patients diagnosed with moderate or severe OSA were prescribed CPAP based on standard medical criteria.

### 2.4. Statistical Analysis

Participants were classified into two groups: those who used CPAP (due to prescription and tolerance of the device) and those who did not (because of lack of prescription in mild OSA or lower tolerance). The median, minimum, and maximum were calculated for the demographic data of each group, while the mean and standard deviation were calculated for neuropsychological test’s parameters. Differences in neuropsychological test scores were assessed by subtracting baseline values from follow-up scores (e.g., Δ MMSE = 12 Months MMSE − Baseline MMSE).

The normality of the data was assessed with Shapiro–Wilk test. Demographic features were compared between CPAP and non-CPAP groups using T-tests and Fisher’s exact tests, as appropriate, based on the normality. Longitudinal changes in CDR were assessed using Stuart-Maxwell test. Longitudinal changes in the other neurocognitive scores were analyzed using both Spearman and Pearson correlation tests for the full sample. The effect of CPAP treatment in the cognitive progression between the baseline and 12-month follow-up (Δ CDR, Δ MMSE, Δ RBANS Total and Δ RBANS sub-domains) was assessed using Generalized Linear Models (GLM) with baseline cognitive outcomes and AHI as covariates. Using GLM, the association among PSG variables and the cognitive variables was also evaluated, with the baseline results of the neuropsychological tests and CPAP as covariates. All statistical analyses were conducted in RStudio (version 4.4.2). The results were considered statistically significant when the *p*-value was lower than 0.05 and the correlation coefficient (r) was higher than 0.3.

## 3. Results

### 3.1. Demographic Description of the CPAP and Non-CPAP Groups

**Figure 1:**
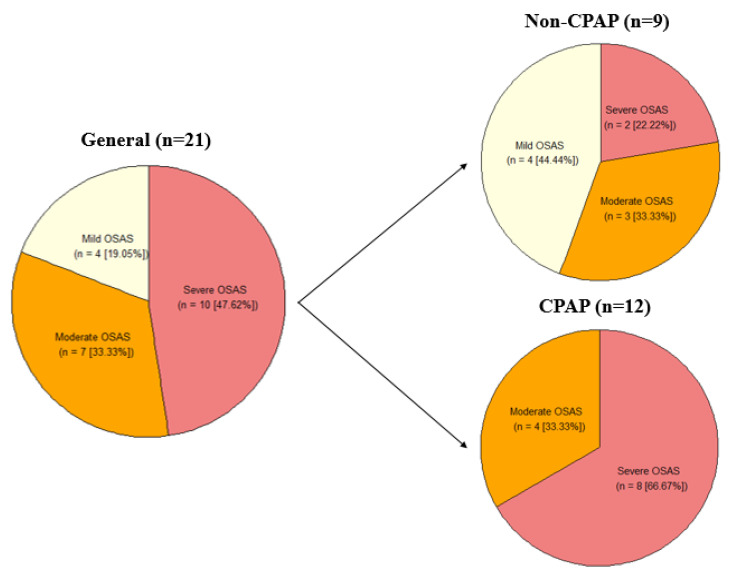
OSA severity in the full cohort and within the CPAP and non-CPAP groups. Representation of the quantity (n) and percentage (%) of participants with Mild, Moderate, and Severe OSA. OSA, Obstructive Sleep Apnea.

A total of 21 patients with MCI were included in this study. The median age of the sample was 77 years (60–81), and 71% (*n* = 15) were female. All patients were diagnosed with some OSA degree and accordingly, they were prescribed CPAP. 57% (*n* = 12) of patients were compliant in CPAP use (≥4 h per night on at least 70% of nights), based on objective device-recorded usage data downloaded from CPAP machines [22]. No statistically significant differences were found in sex and education between CPAP users and non-users (Table 1), but statistically significant differences were identified in age (*p*-value = 0.010), the CPAP group being younger than the non-CPAP group (Figure 1).

### 3.2. CPAP Results

#### Longitudinal Cognitive Status

When focusing on the full cohort included in the current study, statistically significant decline in neurocognitive performance at the 12-month follow-up compared with the baseline assessment were identified in CDR (*p* = 0.223), MMSE (*p* = < 0.001), RBANS Total (*p* = < 0.001), RBANS IMI (*p* = < 0.001), RBANS LL (*p* = < 0.001), RBANS SM (*p* = 0.001), RBANS SF (*p* = < 0.001), RBANS ATI (*p* = < 0.001), RBANS DDS (*p* = 0.005), RBANS C (*p* = < 0.001), RBANS DMI (*p* = 0.003), RBANS SR (*p* = 0.002) and RBANS FR (*p* = 0.003). On the other hand, RBANS LO (*p* = 0.031), RBANS LNGI (*p* = < 0.001), RBANS PN (*p* = 0.016), and RBANS LR (*p* = 0.003) presented better performance at the 12-month follow-up compared with the baseline (Table 2).

When comparing the difference between 12-month follow-up and baseline results in the CPAP and non-CPAP groups, a statistically significant difference was found in Δ MMSE, with lower cognitive decline in the CPAP group (*p* = 0.016). Statistically significant differences were also identified in Δ RBANS Total (*p* = 0.028), Δ RBANS FC (*p* = 0.010), and Δ RBANS SF (*p* = 0.045), with cognitive improvement in the CPAP group (Table 3).

### 3.3. Effect of Sleep in Cognitive Progression

The association between sleep parameters measured at baseline and cognitive progression was evaluated. Only cognitive variables that changed between baseline and 12-month follow-up (Table 2) were evaluated. To ensure that the results were not influenced by the use of CPAP, the use of this device was considered as a covariate for those analyses where a cognitive variable in a previous analysis without covariates was associated with CPAP. Although both age and sex are known to influence sleep architecture and glymphatic function, they were not included as covariates in the models: sex was excluded due to the absence of significant group differences, and age, despite differing between CPAP and non-CPAP groups, did not significantly predict cognitive decline in further analyses.

From the different sleep stages (Table 4), N2 duration showed a significant negative correlation with Δ MMSE, while N3 had a significant positive correlation with Δ MMSE and Δ RBANS FR. REM duration was associated with better cognitive progression measured with Δ RBANS IMI and Δ RBANS LL. From the other sleep parameters, total sleep time showed a significant positive correlation with Δ RBANS IMI, Δ RBANS LL, Δ RBANS SM, Δ RBANS SF, Δ RBANS C, and Δ RBANS SR. Sleep efficiency was positively correlated with Δ RBANS LL, Δ RBANS SF, and RBANS C, while wakefulness had a significant negative correlation with Δ RBANS LL, Δ RBANS SF, and Δ RBANS C. About the sleep position variables, supine time had a significant negative correlation with Δ MMSE, Δ RBANS LO, and Δ RBANS DDS, and right-side time had a significant positive correlation with Δ MMSE and Δ RBANS LO. All these results were independent from the use of CPAP.

## 4. Discussion

In this study, we reported a positive clinical effect of CPAP on cognitive progression in specific cognitive domains. Furthermore, we show an association between baseline sleep patterns, including N2 and N3 time, supine and right-side time, with cognitive progression at 12-month follow-up.

### 4.1. Cognitive Progression in the Full Cohort

The full cohort showed a decline in almost all significant cognitive variables, which is expected in patients with MCI or AD and OSA. Participants improved their scores in RBANS LO, RBANS LNGI, RBANS PN, and RBANS LR. RBANS LO and RBANS LR variables show a cognitive improvement in the CPAP group, while a worsening in non-CPAP group, indicating that this may be attributed to the positive effects of CPAP therapy. For the other two improved variables, RBANS LNGI and RBANS PN, improved scores were greatest in the CPAP group, but the cognition scores for both CPAP and non-CPAP groups improved. This is not consistent with the current literature and with what we expected (cognitive decline in the non-CPAP group, at least) [23]. Potential reasons for unexpected outcomes, such as improved cognitive scores, could be associated with the small size of the full cohort (*n* = 21).

### 4.2. Impact of the CPAP on Cognitive Progression

The positive effect of CPAP on cognitive progression was reported in previous studies. CPAP use was linked with a significant t-tau and t-tau/Aβ_42_ ratio reduction in plasma of OSA patients in a 3-month follow-up [24]. This was translated with an Aβ increase in CSF, which has also been correlated with a decreased brain deposition [25] in a 1-year study with a patient with OSA and subjective cognitive impairment, obtaining the normalization of the cerebral Aβ dynamics and t-tau/Aβ_42_ and Aβ_42_/Aβ_40_ ratios [26]. Previous studies characterized one of the main features of OSA, intermittent hypoxia during sleep, as one of the causes of cognitive decline before AD symptomatology [27,28,29]. During sleep, the dysfunction of the glymphatic system has been implicated as a mechanism for accumulation of Aβ in the brain [30]. This could be related to the cognitive worsening that was obtained in MMSE scores in patients with OSA [31], as well as in other domains (memory, attention, visuospatial, language, etc.) [32]. Other studies also observed slow cognitive decline or cognitive improvement in patients with OSA and AD when treated with CPAP [33,34,35].

Our results are consistent with these studies, obtaining that CPAP use was significantly associated with slower decline in Δ MMSE and an improvement in Δ RBANS Total, as well as an improvement in Δ RBANS FC and Δ RBANS SF sub-domains in a 12-month follow-up study in patients with OSA and MCI due to AD. Importantly, CPAP use did not provoke the decline in Δ MMSE; rather, the decline reflects the natural course of MCI/AD, in which neurodegeneration progresses under optimal treatment. The role of CPAP appears to be in attenuating the speed of this decline, likely through improvements in sleep quality, increased oxygenation, and reduced neuroinflammation [36]. By mitigating OSA-related cognitive stressors, CPAP may preserve certain cognitive functions for longer, even though overall decline is still expected due to the underlying pathology [37].

### 4.3. Effect of Sleep Parameters on Cognitive Progression

The relationship between sleep variables, CPAP therapy, and cognition has been investigated in multiple studies in patients with OSA. However, examining it in patients with OSA and MCI could provide important insights into its appropriate use in patients with cognitive decline.

NREM N2, N3, and REM sleep are critical for cognitive health. N2 was longer in OSA patients and shorter in control and OSA-CPAP groups [38,39]. NREM N3 sleep is the main stage in which the glymphatic system is effective [40]. The impact of this enhancement is an improved clearance of metabolic products in the brain, including Aβ, generating less deposition of this protein and improved cognition when this stage is prolonged. Shorter REM is associated with lower performance in neuropsychological tests [41,42]. The results obtained in our study are consistent with this literature for NREM N2, in which longer stages have been associated with cognitive worsening in Δ MMSE, as well as for NREM N3, with longer stages correlated with cognitive improvement in Δ MMSE and Δ RBANS FR, and REM sleep, with longer duration associated with better cognition in Δ RBANS IMI and Δ RBANS LL.

Total sleep time and sleep efficiency also play significant roles in maintaining cognitive health. Studies have demonstrated that higher total sleep time would facilitate the repetition of sleep cycles, allowing NREM N3 to be longer [43]. Other studies have also described that sleep duration shorter or longer than certain limits, generally described as <5 h and >9 h in 70-year-old participants, would be associated with worsening cognition with a quadratic trend that cannot be well-established with linear models [44]. There are disruptions too, like wakefulness that can affect sleep efficiency, which in turn can further fragment sleep when it is longer, generating poor cognition [45]. This information supports our results for total sleep time, sleep efficiency, and wakefulness as longer total sleep time was associated with better cognition in Δ RBANS IMI, Δ RBANS LL, Δ RBANS SM, Δ RBANS SF, Δ RBANS C, and Δ RBANS SR, higher sleep efficiency was correlated with cognitive improvement in Δ RBANS LL, Δ RBANS SF, and Δ RBANS C, and longer wakefulness with cognition worsening in Δ RBANS LL, Δ RBANS SF, and Δ RBANS C.

Right-side time and supine time are essential to understanding the sleep position importance that have been studied by other research groups. In humans, the “Starling resistor” mechanism in normal conditions prevents the overdrainage of cranial venous blood in supine sleeping position. In OSA, this mechanism is altered because a pressure airway drops below a critical value, generating the collapse of the upper airway [46,47]. This collapse activates a defense mechanism based on a choking feeling that may disrupt sleep in patients, affecting their cognition in chronic events [48]. Lower CPAP can maintain the upper airway open easier in lateral than in supine position, helping to prevent this aforementioned collapse [49]. A possible reason for this could be that the collapse is easier in the supine position than in the lateral position, due to the gravitational effect. This collapse is associated with an increase in apneas and respiratory disruptions when sleeping [50], worsening cognition. These studies are consistent with the result obtained for supine time, in which longer time is associated with cognitive decline in Δ MMSE, Δ RBANS LO, and Δ RBANS DDS.

When sleeping in lateral position, it was observed that right position generates an increase in vagal tone and a reduction in sympathetic tone stronger than in left position [51]. It leads to the highest vagal modulation [52], where fibers provide parasympathetic innervation to the atria and sinoatrial node, while the left vagal nerve just innervates the ventricles [53]. Vagus Nerve Stimulation (VNS), an FDA-approved treatment for epilepsy and depression among others, has been associated with increased penetration of CSF into the brain, increasing brain waste clearance in mice [54]. This information could be the reason why longer right-side time is associated with cognitive improvement in Δ MMSE and Δ RBANS LO in our study, thus, sleeping in a right-side position would cause a stronger activation of parasympathetic tone in heart and affect CSF penetrance.

The greater vagal tone and reduced sympathetic tone observed during right lateral sleep may be explained by neurochemical shifts involving ACh and NE, key neurotransmitters regulating parasympathetic and sympathetic activity, respectively. During SWS, NE release from the locus coeruleus is significantly reduced. This reduction in NE lowers sympathetic drive, allowing the expansion of interstitial and perivascular spaces in the brain, which increases CSF flow through the glymphatic system. This enhanced CSF penetration facilitates more effective clearance of metabolic waste products such as Aβ, which is crucial for cognitive health [55].

Although ACh levels are generally low during SWS to permit synchronized slow oscillations in neuronal activity, parasympathetic/vagal activation mediated by ACh in the periphery helps regulate vascular tone and cerebral perfusion. The right lateral sleep position appears to boost parasympathetic activity more strongly than other positions, as evidenced by increased vagal tone and decreased sympathetic output. This enhanced parasympathetic influence may further support optimal blood flow and maintain vascular dynamics that indirectly facilitate glymphatic clearance [55].

In the overall literature, it has been described that sleep deprivation evidences a decrease in comprehension, attention, language, and memory [56,57,58,59]. This could explain why we have obtained these results, affecting longer sleep time and greater sleep efficiency in MMSE and some attention, language, and memory RBANS sub-domains. Regarding the sleep position, it has been observed in our results that these positions could also have consequences. Thus, supine position had a negative effect on MMSE and RBANS sub-domains related to attention and memory, while right-side position showed cognitive improvement in MMSE and a memory RBANS sub-domain.

### 4.4. Glymphatic System and Sleep-Cognition Relationships

The glymphatic system, active primarily during NREM N3, plays a critical role in clearing neurotoxic waste such as Aβ from the brain. Sleep disturbances, especially disruptions in N3 sleep, can impair this clearance, contributing to Aβ accumulation and subsequent cognitive decline [60]. Our findings of significant correlations between cognitive changes and N2/N3 sleep stages highlight this relationship, where longer N3 promotes cognitive preservation, likely via enhanced glymphatic clearance, while prolonged N2 correlates with decline, possibly reflecting compensatory or fragmented sleep.

Regarding the significant effects observed between MMSE scores and changes in N2 and N3 sleep stages, the statistical significance indicates that specific alterations in sleep architecture are closely linked to global cognitive function. Increased N3 duration aligns with better MMSE outcomes, consistent with its restorative role and glymphatic activation, while increased N2 duration might reflect lighter, less restorative sleep and correlates with worse cognition.

Our results are consistent with these studies, obtaining that CPAP use was significantly associated with slower decline in Δ MMSE and an improvement in Δ RBANS Total, as well as an improvement in Δ RBANS FC and Δ RBANS SF sub-domains in a 12-month follow-up study in patients with OSA and MCI due to AD. These results are due to CPAP impact on sleep quality improvement, as well as an increase in oxygenation and a reduction in neuroinflammation. Consequently, better concentration and memory make these two sub-domains to be significantly improved.

### 4.5. Study Limitations

This study has some limitations. Although prospective, it is a pilot study with a reduced sample size (*n* = 21) and an unequal distribution of participants between the CPAP (*n* = 12) and non-CPAP (*n* = 9) groups, which limits statistical power and the generalizability of the findings. Therefore, results should be interpreted cautiously. CPAP adherence was objectively measured using device-derived data and defined as ≥4 h/night on ≥70% of nights, but adherence in AD patients remains challenging, particularly in more advanced stages of the disease. Additionally, V-PSG data were derived from a single-night study, which may not fully capture habitual sleep patterns. For future studies, long-term monitoring methods such as actigraphy could provide more robust insights [61]. Despite these limitations, we have identified significant results that are consistent with previous literature.

## 5. Conclusions

CPAP use in patients with OSA and MCI was associated with slower cognitive decline, particularly in global cognition and specific domains, over 12 months. While CPAP did not stop cognitive deterioration, it appeared to attenuate the rate of decline compared to non-users. Sleep duration and position also emerged as relevant factors in cognitive progression. Larger, adequately powered, and longer-term studies are required to confirm these findings, explore domain-specific effects more comprehensively, determine whether CPAP can meaningfully delay the onset of dementia symptoms in this population, and identify strategies to improve adherence in AD patients.

## Figures and Tables

**Table 1:** CPAP and non-CPAP Demographic Data.

Features	Non-CPAP (*n* = 9)	CPAP (*n* = 12)	*p*-Value
Age, yrs	79 (72–81)	74 (60–78)	**0.010**
Sex			1.000
Females	6 (66.67%)	9 (75.00%)	
Males	3 (33.33%)	3 (25.00%)	
Education, yrs	12 (5–18)	9 (2–20)	0.327

*p*-value < 0.05 is highlighted in **bold**. **Abbreviation**: Yrs, Years.

**Table 2:** Neurocognitive Performance of all Subjects at Baseline and 12 Months Follow-Up.

Neurocognitive Test/Assessment	Score T0 (Baseline) (Mean ± SD)	Score T1 (12 Months) (Mean ± SD)	*p*-Value
CDR	0.64 ± 0.36	0.71 ± 0.37	0.223
MMSE	22.67 ± 5.35	20.71 ± 5.51	**<0.001**
RBANS Total	66.24 ± 14.77	64.24 ± 15.48	**<0.001**
RBANS IMI	65.05 ± 15.50	61.29 ± 15.25	**<0.001**
RBANS LL	17.90 ± 5.51	16.50 ± 6.07	**<0.001**
RBANS SM	7.24 ± 4.33	6.33 ± 4.82	**0.001**
RBANS VSPI	85.89 ± 19.38	87.95 ± 17.08	0.056
RBANS FC	16.65 ± 2.66	17.10 ± 1.92	0.365
RBANS LO	13.00 ± 4.24	13.05 ± 4.05	**0.031**
RBANS LNGI	74.21 ± 19.12	77.68 ± 16.45	**<0.001**
RBANS PN	7.84 ± 2.14	8.58 ± 1.71	**0.016**
RBANS SF	11.71 ± 5.58	11.38 ± 5.16	**<0.001**
RBANS ATI	69.42 ± 18.14	69.11 ± 18.59	**<0.001**
RBANS DDS	4.86 ± 1.28	4.48 ± 1.33	**0.005**
RBANS IDS	2.82 ± 2.83	4.47 ± 1.55	0.768
RBANS C	21.00 ± 14.15	20.00 ± 14.86	**<0.001**
RBANS DMI	64.75 ± 19.35	57.30 ± 19.49	**0.003**
RBANS LR	1.10 ± 1.67	1.33 ± 2.20	**0.003**
RBANS LRe	14.90 ± 4.38	14.50 ± 2.70	0.349
RBANS SR	2.57 ± 2.77	1.86 ± 2.41	**0.002**
RBANS FR	5.86 ± 5.08	5.48 ± 5.71	**0.003**

*p*-value < 0.05 is highlighted in **bold**. **Abbreviations**: SD, Standard Deviation; MMSE, Mini-Mental State Examination; RBANS, Repeatable Battery for the Assessment of Neuropsychological Status; LL, List Learning; SM, Story Memory; IMI, Immediate Memory Index; FC, Figure Copy; LO, Line Orientation; VSPI, Visuospatial Index; PN, Picture Naming; SF, Semantic Fluency; LNGI, Language Index; DDS, Direct Digit Span; IDS, Indirect Digit Span; C, Coding; ATI, Attention Index; LR, List Recall; LRe, List Recognition; SR, Story Recall; FR, Figure Recall; DMI, Delayed Memory Index.

**Table 3:** Groups’ Neurocognitive Performance Differences at 12 Months Follow-Up Regarding Basal Diagnosis.

Neuropsychological Test/Assessment	Non-CPAP (*n* = 9)	CPAP (*n* = 12)	*p*-Value	Confidence Interval (95%)	
	T1 (12 Months)–T0 (Baseline) (Mean ± SD)	T1 (12 Months)–T0 (Baseline) (Mean ± SD)		Lower Bound	Upper Bound
CDR	0.06 ± 0.17	0.08 ± 0.19	0.838	−0.214	0.173
MMSE	−3.78 ± 2.54	−0.58 ± 3.42	**0.016**	**1.071**	**6.967**
RBANS Total	−6.38 ± 7.73	1.89 ± 9.09	**0.028**	**2.320**	**20.104**
RBANS IMI	−4.78 ± 9.96	−3.00 ± 14.52	0.229	−4.153	18.659
RBANS LL	−2.56 ± 4.10	−0.45 ± 4.91	0.589	0.160	8.700
RBANS SM	−0.78 ± 2.91	−1.00 ± 4.59	0.495	−2.480	5.217
RBANS VSPI	−6.11 ± 14.83	9.40 ± 20.59	0.119	−2.529	29.632
RBANS FC	−1.11 ± 2.09	1.73 ± 2.97	**0.010**	**0.820**	**4.275**
RBANS LO	−0.33 ± 2.74	0.40 ± 4.81	0.988	−3.812	3.756
RBANS LNGI	2.44 ± 12.27	4.40 ± 15.68	0.177	−3.563	22.073
RBANS PN	0.67 ± 1.41	0.80 ± 1.99	0.643	−1.129	1.846
RBANS SF	−1.67 ± 2.69	0.67 ± 4.10	**0.045**	**0.348**	**7.167**
RBANS ATI	−1.38 ± 6.80	0.45 ± 8.49	0.834	−8.081	10.044
RBANS DDS	−0.33 ± 0.87	−0.42 ± 0.79	0.961	−0.893	0.849
RBANS IDS	2.00 ± 3.16	1.33 ± 4.27	0.151	−2.719	0.338
RBANS C	−3.38 ± 6.07	0.73 ± 6.31	0.135	−1.379	12.846
RBANS DMI	−18.00 ± 13.87	1.18 ± 15.52	0.064	0.167	27.458
RBANS LR	−0.44 ± 0.88	0.75 ± 2.18	0.326	−0.658	0.304
RBANS LRe	−2.67 ± 3.43	1.45 ± 4.95	0.145	−0.584	4.757
RBANS SR	−0.78 ± 1.09	−0.67 ± 1.87	0.211	−0.466	2.298
RBANS FR	−0.67 ± 4.09	−0.17 ± 4.93	0.802	−3.980	5.165

*p*-value < 0.05 is highlighted in **bold**. **Abbreviations**: SD, Standard Deviation; MMSE, Mini-Mental State Examination; RBANS, Repeatable Battery for the Assessment of Neuropsychological Status; LL, List Learning; SM, Story Memory; IMI, Immediate Memory Index; FC, Figure Copy; LO, Line Orientation; VSPI, Visuospatial Index; PN, Picture Naming; SF, Semantic Fluency; LNGI, Language Index; DDS, Direct Digit Span; IDS, Indirect Digit Span; C, Coding; ATI, Attention Index; LR, List Recall; LRe, List Recognition; SR, Story Recall; FR, Figure Recall; DMI, Delayed Memory Index.

**Table 4:** Relationships Between Baseline V-PSG and Cognitive Progression.

		Sleep Stages			Sleep Quality			Sleep Position	
		N2	N3	REM	TST	SE	W	ST	RST
Δ MMSE	*p*-value	**0.038**	**0.022**	0.879	0.856	0.673	0.655	**0.023**	**0.011**
	r	**−0.41**	**0.51**	0.14	0.01	0.01	−0.02	**−0.44**	**0.56**
Δ RBANS Total	*p*-value	0.415	0.299	0.766	0.055	0.090	0.094	0.549	0.076
	r	0.19	0.21	0.03	**0.35**	0.29	−0.29	−0.10	**0.35**
Δ RBANS IMI	*p*-value	0.106	0.977	**0.044**	**0.014**	**0.038**	**0.048**	0.420	0.966
	r	**0.32**	−0.17	**0.42**	**0.38**	0.29	−0.27	0.25	−0.12
Δ RBANS LL	*p*-value	0.393	0.628	**0.015**	**0.024**	**0.036**	**0.041**	0.705	0.866
	r	0.18	0.01	**0.53**	**0.40**	**0.36**	**−0.35**	0.12	−0.02
Δ RBANS SM	*p*-value	0.106	0.865	0.294	**0.038**	0.118	0.148	0.329	0.877
	r	**0.35**	−0.17	0.20	**0.39**	0.26	−0.23	0.28	−0.07
Δ RBANS LO	*p*-value	0.928	0.249	0.985	0.214	0.294	0.360	**0.041**	**0.002**
	r	−0.08	0.28	−0.13	0.10	0.07	−0.06	**−0.43**	**0.54**
Δ RBANS LNGI	*p*-value	0.280	0.509	0.682	0.098	0.183	0.194	0.579	0.679
	r	**0.42**	−0.04	−0.27	**0.31**	0.27	−0.26	0.26	−0.03
Δ RBANS PN	*p*-value	0.216	0.683	0.893	0.235	0.379	0.390	0.153	0.410
	r	**0.49**	**−0.32**	−0.19	0.25	0.21	−0.21	**0.46**	**−0.30**
Δ RBANS SF	*p*-value	0.191	0.281	0.266	**0.032**	**0.018**	**0.016**	0.882	0.656
	r	**0.37**	0.09	0.11	**0.33**	**0.39**	**−0.41**	0.12	−0.02
Δ RBANS ATI	*p*-value	0.291	0.625	0.955	0.278	0.456	0.465	0.855	0.615
	r	0.24	0.13	−0.02	0.22	0.15	−0.14	−0.04	0.10
Δ RBANS DDS	*p*-value	0.178	0.154	0.781	0.242	0.328	0.344	**0.014**	0.058
	r	**−0.33**	**0.36**	−0.12	**−0.30**	−0.25	0.24	**−0.53**	**0.39**
Δ RBANS C	*p*-value	0.064	0.348	0.993	**0.020**	**0.016**	**0.014**	0.445	0.937
	r	**0.42**	0.21	−0.02	**0.47**	**0.49**	**−0.50**	0.19	−0.03
Δ RBANS DMI	*p*-value	0.227	0.536	0.759	0.427	0.317	0.300	0.632	0.639
	r	**0.32**	0.03	0.09	0.24	0.29	**−0.31**	−0.04	0.04
Δ RBANS LR	*p*-value	0.196	0.515	0.769	0.191	0.121	0.109	0.084	0.271
	r	**0.32**	0.06	0.09	0.29	**0.35**	**−0.36**	**0.43**	**−0.30**
Δ RBANS SR	*p*-value	0.181	0.360	0.079	**0.019**	0.055	0.073	0.776	0.216
	r	**0.40**	−0.10	**0.45**	**0.51**	**0.40**	**−0.37**	0.24	0.05
Δ RBANS FR	*p*-value	0.328	**0.035**	0.473	0.549	0.253	0.196	0.273	0.207
	r	0.25	**0.46**	−0.11	0.13	0.25	−0.28	−0.24	**0.30**

*p*-value < 0.05 and |r| > 0.30 are highlighted in **bold**. **Abbreviations**: MMSE, Mini-Mental State Examination; RBANS, Repeatable Battery for the Assessment of Neuropsychological Status; LL, List Learning; SM, Story Memory; IMI, Immediate Memory Index; LO, Line Orientation; PN, Picture Naming; SF, Semantic Fluency; LNGI, Language Index; DDS, Direct Digit Span; C, Coding; ATI, Attention Index; LR, List Recall; LRe, List Recognition; SR, Story Recall; FR, Figure Recall; DMI, Delayed Memory Index; N2: NREM Stage 2 (min); N3: NREM Stage 3 (min), REM: Rapid-Eye Movement (min); TST: Total Sleep Time (min); SE: Sleep Efficiency (%); W: Wakefulness (min); ST: Supine Time (min); RST: Right-Side Time (min).

## Data Availability

The original contributions presented in this study are included in the article. Further inquiries can be directed to the corresponding author(s).
